# Childhood cigarette smoking is associated with health-related quality of life in older US adults

**DOI:** 10.18332/tid/204009

**Published:** 2025-07-23

**Authors:** Jenny E. Ozga, James D. Sargent, Alexander W. Steinberg, Zhiqun Tang, Cassandra A. Stanton, Laura M. Paulin

**Affiliations:** 1 Behavioral Health and Health Policy, Westat, Rockville, United States; 2 Department of Pediatrics, Geisel School of Medicine at Dartmouth, Hanover, United States; 3 Department of Biomedical Data Science, Geisel School of Medicine at Dartmouth, Hanover, United States; 4 Department of Medicine, University of Washington School of Medicine, Seattle, United States; 5 Section of Pulmonary and Critical Care, Department of Medicine, Dartmouth-Hitchcock Medical Center, Lebanon, United States

**Keywords:** cigarette smoking, health-related quality of life, childhood smoking

## Abstract

**INTRODUCTION:**

Childhood smoking onset is associated with chronic obstructive pulmonary disease (COPD), independent of current smoking and smoking history. Its association with lower quality of life has not been tested. We examined the association between childhood smoking and measures of global health among older US adults.

**METHODS:**

This study involved a cross-sectional survey of US adults ≥40 years (n=7056) from Wave 5 (2019) of the Population Assessment of Tobacco and Health Study. Ever smokers were asked when they began smoking regularly. Weighted multivariable analysis assessed onset of regular smoking at age of <15 years as a risk factor for lower Patient-Reported Outcomes Measurement Information System Global Physical and Mental Health (GPH and GMH) scores, adjusting for current smoking, smoking intensity, cigarette pack-years, secondhand smoke exposure, and other covariates. Sensitivity analysis added smoking-related disease comorbidities (e.g. COPD).

**RESULTS:**

Sociodemographics were reflective of the US population aged ≥40 years, with 6.8% reporting childhood smoking and 16.9% current smoking. Mean (SD) for GPH and GMH were 14.8 (3.1) and 14.5 (3.8), respectively. In the multivariable analysis, compared to onset smoking at an older childhood age, GPH and GMH were significantly lower for those with early onset of smoking (-4.27%; 95% CI: -6.52 – -1.97 and -3.34%; 95% CI: -6.08 – -0.52; respectively). Global health was also negatively associated with current smoking, higher pack-years, and secondhand smoke exposure. The association between childhood smoking and GPH (but not GMH) remained significant after further adjustment for disease mediators.

**CONCLUSIONS:**

Childhood smoking was independently associated with lower quality of life among adults aged ≥40 years. Adverse effects of smoking on development during adolescence may have implications that extend beyond associations with chronic disease.

## INTRODUCTION

Lifetime cigarette smoking has profound effects on health, affecting every organ in the body, and is causally associated with dozens of diseases and millions of deaths worldwide each year^1^. Cigarette smoking is also associated with health-related lower quality of life (HRQOL)^2^, even among the young^3^. Among measures of cigarette smoking, current smoking has been associated with lower HRQOL in many studies^2^, with heavier smoking^4^ and longer smoking history^5^ also associated with lower HRQOL. Among older adults with smoking-related disease, those who continued to smoke had lower HRQOL compared to those who quit smoking^6-8^, and smokers who successfully quit reported gains in HRQOL over time^9^. Finally, even among adults who have never smoked, HRQOL was lower for those exposed to secondhand smoke^10,11^.

Studies have drawn attention to the association between childhood smoking and respiratory disease^12^. In one recent study, those with smoking onset at an age of <15 years was associated with chronic obstructive pulmonary disease (COPD), net current smoking, pack-years of smoking, and secondhand smoke exposure^13^, raising concerns that smoke-related effects on lung development during adolescence^14^ could have long-term consequences beyond risk conferred by established factors such as pack-years of smoking. Another found that COPD patients whose smoking began during the alveolar development phase (<24 years) had poorer lung function, more exacerbations, needed more intensive medical treatment, and had lower survival compared to later onset smokers^15^. This body of research raises the question of whether childhood smoking affects HRQOL, independent of other established smoking risk factors.

The present cross-sectional study examines whether childhood smoking is associated with measures of physical and mental global health in a representative sample of US older adults. We also compare the size of the association with other established smoking risk factors. In a sensitivity analysis, we test whether the association persists after further adjustment for causal pathway mediators, like COPD.

## METHODS

### Study participants

We analyzed cross-sectional data from wave 5 (W5, 2018019) for the Population Assessment of Tobacco and Health (PATH) Study. The PATH Study is a national cohort survey of US adults (aged ≥18 years). Surveys were conducted in respondents’ households using computer-assisted self-interviews. The present study used the W1-W5 Adult Restricted Use Files and was limited to adults aged ≥40 years at W1 (n=14335), when many smoking-related diseases become manifest. Of these, 4209 (22.7%) were lost to follow-up from W1-W5 and 3070 had missing data for one or more items in the survey (see Supplementary file Figure 1 for flow diagram).

### Primary outcome patient-reported outcomes measurement information system (PROMIS) global physical and mental health

PROMIS includes >300 measures of physical, mental, and social health for use with the general population^16^. The PATH Study employed the PROMIS Global-10 sub-questionnaire, which is used to measure global health. Our outcome included a four-item Global Physical Health (GPH) subscale and Global Mental Health (GMH) subscale (both scored 4–20)^17^. These scales were validated by comparing similar domains in the more comprehensive EuroQol-5D (EQ-5D) instrument^18^. Higher scores indicate a better HRQOL. In our PATH Study analytic sample, both subscales had acceptable internal reliability (Cronbach’s alpha values of 0.72 and 0.82 for GPH and GMH, respectively).

### Main exposure variable

People who had ever smoked 100 cigarettes in their entire life were asked: ‘How old were you when you first started smoking regularly?’. In a study of childhood smoking and COPD^13^, there was little evidence of an association between age of smoking onset and COPD for persons who began smoking after 20 years, but COPD prevalence increased linearly for adults who smoked during childhood, approaching 30% for those who began smoking at 10 years of age (Supplementary file Figure 2).

### Cigarette smoking covariates

Among adults who had ever smoked, current smoking was assessed by asking: ‘Do you now smoke cigarettes every day, some days, or not at all?’. Based on whether they reported smoking >100 cigarettes in their lifetime, this variable was recoded as never (<100 cigarettes lifetime); former (>100 lifetime, not at all now), and current (>100 lifetime), some days; or current, every day. Adults who formerly smoked were asked how many years since they quit smoking cigarettes and usual amount smoked, used to determine duration and pack-years of smoking. Smoking intensity (‘On average how many cigarettes do you now smoke per day’) was used to calculate pack-years for adults who currently smoke. Secondhand smoke exposure was included as a continuous measure based on responses to the question: ‘In the past 7 days, number of hours that you were in close contact with others when they were smoking?’.

We selected other covariates based on previous studies that assessed the relationship between tobacco consumption and PROMIS. Socioeconomic factors are well-established quality of life indicators. Although many previous studies have included tobacco-related disease items as covariates in studies of tobacco and QOL, we felt that that was theoretically unsound, because the pathway from tobacco use to disease to reduced health-related QOL is a well-established causal chain.

### Statistical analysis

We first created a limited dataset that included the GPH and GMH outcome measures, the main exposure variable (childhood smoking), and covariates (smoking status, pack-years, secondhand smoke exposure, age, sex, race, annual income, education level, and residence geographical location). In this limited dataset, 4.7% of values were missing, and we performed listwise deletion to exclude respondents who were missing data on the outcome measures, the main exposure variable, or any covariates (Supplementary file Figure 1). We then fit lowess smoothed curves for the association between age of smoking onset and mean GPH and GMH, to determine if there was a similar dose-response curve to COPD prevalence (Supplementary file Figure 2).

Neither GPH nor GMH outcomes (nor their residuals) were normally distributed (Shapiro-Wilk tests with p<0.05). We used non-parametric Kruskal-Wallis tests to examine differences in mean GPH and GMH based on age of cigarette onset category. Significant Kruskal-Wallis tests were followed up with pairwise Mann-Whitney U tests with Bonferroni correction, which resulted in a significance cutoff of p<0.017. Given that both outcome distributions were strictly positive and skewed to the left, we further used cross-sectional weighted gamma-distributed generalized linear models (GLMs) with a log link to examine the bivariable relations between childhood smoking and measures of global health as well as the covariates. Because gamma-distributed GLM assumes that data are strictly positive and skewed to the right (rather than skewed to the left), we first calculated the inverse of each outcome (1/GPH or 1/GMH) prior to conducting regression models. We then estimated the independent association between childhood smoking and GPH and GMH separately using multivariable weighted gamma-distributed GLMs, adjusting for covariates as described above. For ease of interpretation in terms of the original outcome scales, coefficients from regression models were inverse exponentiated and converted to percentages. Percent differences in the outcomes and their corresponding 95% confidence intervals (CIs) are reported as results, with positive values indicating an increase in the outcome and negative values indicating a decrease in the outcome relative to the reference category.

Current and former smoking were combined for the reference category to avoid multicollinearity with the childhood smoking variable in multivariable analysis. Analyses were weighted using W5 all-waves survey weights, which included full sample and 100 replicate weights, to produce nationally representative estimates. The W5 all-waves weights accounted for oversampling at W1, enrichment of the sample at W4 and attrition from W1-W5, obtaining estimates for the US population. Variances were computed using the balanced repeated replication (BRR) method with Fay’s adjusted set to 0.3 to increase estimate stability. Details on how replicate weights were conducted by the PATH Study are in the PATH Study’s Restricted Files User Guide. All analyses were two tailed and the p-value for statistical significance was 0.05. This is a complete case analysis and did not use advanced methods to account for item missingness. Analyses were conducted using Stata version 18.5.

We did not adjust our main analysis for smoking-related diseases, because in theory, these variables were mediators of the causal pathway from cigarette smoke exposure to poorer HRQOL. In a sensitivity analysis, we tested whether there were residual direct associations between measures of cigarette smoke exposure and global health measures after further adjustment for self-reported COPD and a comorbidity index. In addition to adjusted estimates, we also report on the proportion of effect mediated and ratio of total to direct effect for childhood smoking after additional adjustment for COPD and other comorbidities. The disease comorbidity index score has been used in prior studies to capture important contributors to morbidity in COPD patients, which included diabetes, any cancer, congestive heart failure, high blood pressure, heart attack, osteoporosis, peripheral vascular disease, stroke, ulcer(s), obesity, or high cholesterol (range 0–11; Cronbach's alpha=0.59)^19^. Respondents with missing data for self-reported COPD (n=12) and the comorbidity index (n=43) were excluded from this sensitivity analysis. Finally, we substituted smoking duration for cigarette pack-years in an additional sensitivity analysis as previous research has found that smoking duration may be a better indicator of smoking history than pack-years of smoking^20^.

## RESULTS

### Sample description

The sample (n=7056) was largely representative of the US population aged ≥40 years (Table 1), with higher percentages of females (54.5%), with Black adults representing 11.3%, and with 24.5% having annual income (US$) of <25000. Regarding tobacco use, 6.8% smoked during childhood, and 16.9% currently smoked. The weighted mean (SD) for pack-years of smoking among adults who ever smoked was 32.9 (48.0), and hours of exposure to secondhand smoke per week was 3.6 (13.4). Mean (SD) for GPH and GMH were 14.8 (3.1) and 14.5 (3.8), respectively (Supplementary file Table 1 gives percentile distribution).

**Table 1:** Characteristics of the sample, weighted means and percentages, a cross-sectional analysis of Wave 5 (2018–2019), Population Assessment of Tobacco and Health Study for adults aged ≥40 years (N=7056), restricted use files analyzed 2024 (estimates reflect percent difference compared to reference)

*Characteristics*	*Total* *n (%)*	*Global health* *Mean (SD)*	
		*Physical*	*Mental*
**Tobacco risk factors**			
**Age of childhood smoking onset** (years)			
Never	2644 (55.2)	15.9 (2.2)	15.6 (2.5)
<15	784 (6.8)	14.0 (4.2)	13.8 (4.5)
≥15	3628 (38.0)	14.9 (3.4)	14.6 (3.8)
**Current smoking**			
Never	2644 (55.2)	15.9 (2.2)	15.6 (2.5)
Former	1668 (28.0)	15.3 (2.6)	15.1 (2.8)
Current	2744 (16.9)	14.0 (4.6)	13.4 (5.2)
**Pack-years**, mean	22.0	-	-
**Secondhand smoke exposure** (hours/week), mean	3.6	-	-
**Sociodemographic factors**			
**Age** (years)			
40–49	2122 (24.9)	15.8 (3.0)	15.2 (3.5)
50–59	2131 (26.4)	15.4 (3.1)	14.8 (3.6)
60–69	1708 (24.8)	15.3 (2.9)	15.1 (3.1)
70–79	835 (16.8)	15.2 (2.5)	15.5 (2.5)
≥80	260 (7.1)	14.6 (2.0)	14.9 (1.9)
**Sex**			
Female	3794 (54.5)	15.3 (3.0)	15.0 (3.3)
Male	3262 (45.5)	15.5 (2.7)	15.2 (3.1)
**Race**			
White	5416 (80.7)	15.4 (2.8)	15.1 (3.1)
Black	1106 (11.3)	14.9 (3.4)	14.9 (3.6)
Other or multiracial	534 (8.0)	15.7 (2.9)	15.5 (3.1)
**Annual income** (1000 US$)			
≥100	1405 (23.0)	16.6 (2.0)	16.2 (2.6)
50 to <100	1665 (25.4)	15.9 (2.3)	15.6 (2.7)
25 to <50	1479 (20.2)	15.0 (2.9)	14.7 (3.3)
<25	2156 (24.5)	13.9 (3.7)	13.8 (3.9)
Refused/don’t know	351 (6.9)	15.4 (2.4)	15.3 (2.4)
**Education level**			
<HS or GED	1361 (15.4)	14.0 (3.5)	13.8 (3.8)
High school diploma	1396 (22.8)	14.8 (2.8)	14.8 (2.9)
Some college or associate’s	2288 (29.8)	15.3 (2.9)	15.0 (3.2)
Bachelor’s or higher	2011 (32.0)	16.5 (2.2)	16.0 (2.8)
**Residence**			
Urban	5228 (76.6)	15.5 (2.8)	15.1 (3.1)
Non-urban	1828 (23.4)	15.1 (3.1)	14.9 (3.4)

### Dose-response between age of onset for smoking and health-related quality of life

**Figure 1:**
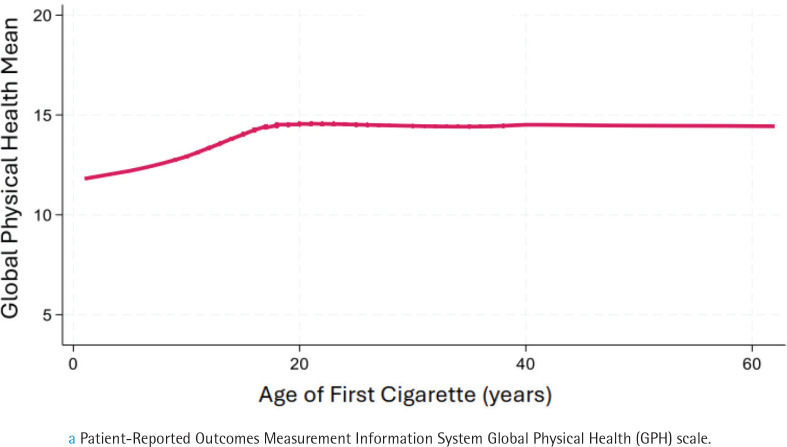
Lowess smoothed curve showing the association between age of first cigarette and mean score on health-related quality of life, focused on physical healtha, a cross-sectional analysis of Wave 5 (2018–2019), Population Assessment of Tobacco and Health Study of adults aged ≥40 years (N=7056) (restricted use files analyzed 2024)

**Figure 2:**
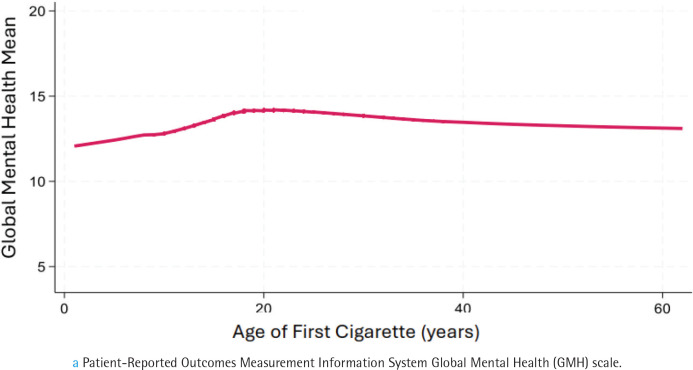
Lowess smoothed curve showing the association between age of first cigarette and mean score on health-related quality of life, focused on mental healtha, a cross-sectional analysis of Wave 5 (2018–2019), Population Assessment of Tobacco and Health Study of adults aged ≥40 years (N=7056) (restricted use files analyzed 2024)

The relation between GPH (Figure 1) and GMH (Figure 2) and age of smoking onset was similar in form to the COPD association, with scores declining at age <18 years. These data justified retaining a cutoff level of 15 years for defining childhood smoking for our investigation of its relation to HRQOL. For the analysis, age started smoking regularly was entered as categorical variable with ≥15 years as the reference category.

### Bivariable associations

**Figure 3:**
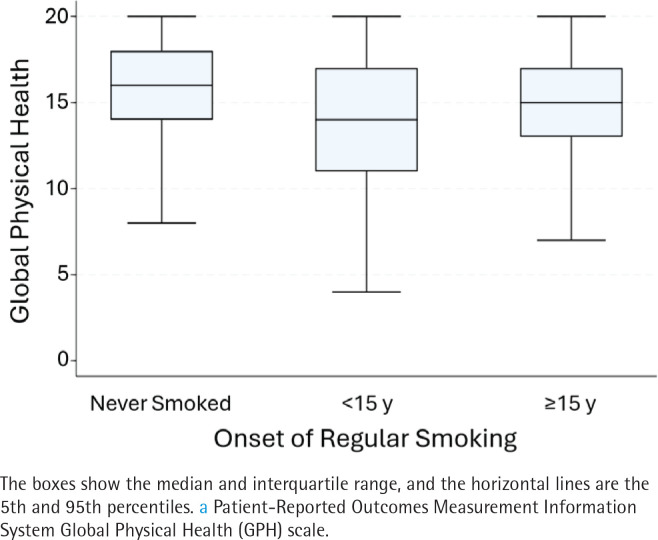
Box plots showing the distribution of the health-related quality of life, focused on physical health^a^, a cross-sectional analysis of Wave 5 (2018–2019), Population Assessment of Tobacco and Health Study of adults aged ≥40 years (N=7056) (restricted use files analyzed 2024)

**Figure 4:**
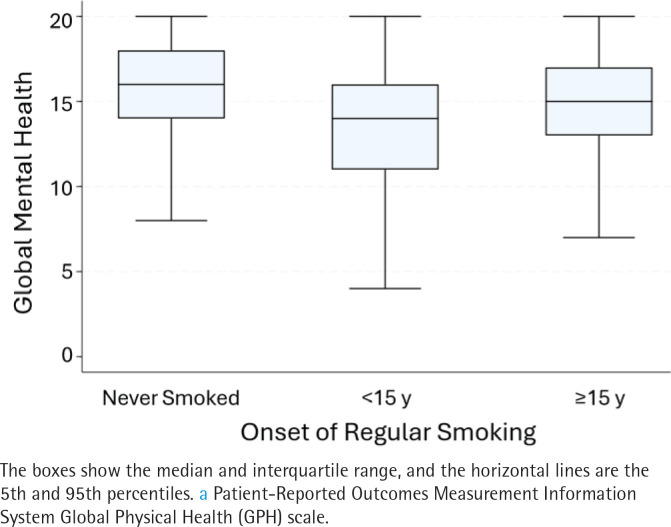
Box plots showing the distribution of the health-related quality of life, focused on mental health^a^, a cross-sectional analysis of Wave 5 (2018–2019), Population Assessment of Tobacco and Health Study of adults aged ≥40 years (N=7056) (restricted use files analyzed 2024)

Adults who smoked during childhood had means that were about one point lower for GPH and GMH compared to those whose smoking onset was at ≥15 years (Table 1), about the same as the difference in means for current versus never/former smoking. Figures 3 and 4 give further details on the distribution for GPH (Figure 3) and GMH (Figure 4) by childhood smoking status. Compared to adults who never smoked, the global health distributions were shifted downwards for both smoking categories, regardless of when smoking started. However, the distributions for childhood smoking showed a more dramatic shift, with medians being about equivalent to the 25th percentile for adults who never smoked. This widening was reflected in a widened interquartile range (IQR) for adults who smoked during childhood (6 for GPH and 5 for GMH) compared to an IQR of 4 for both outcomes in adults who never smoked. The differences in GPH and GMH by childhood smoking status were both highly significant (p<0.0001) by the Kruskal-Wallis and Man Whitney U *post hoc* tests. Table 2 shows unadjusted estimates for each factors’ bivariable association with QOL outcomes; they were consistent with but larger than the multivariable estimates.

**Table 2:** Weighted multivariable gamma-distributed generalized linear model estimates of the association between measures of cigarette smoking and health-related quality of life outcomes^a^, a cross-sectional analysis^b^ of Wave 5 (2018–2019), Population Assessment of Tobacco and Health Study for adults aged ≥40 years (N=7056), restricted use files analyzed 2024 (estimates reflect percent difference compared to reference)

*Variables*	*Global physical health*			*Global mental health*		
	*Percent difference*			*Percent difference*		
	*Unadjusted*	*Adjusted*	*95% CI*	*Unadjusted*	*Adjusted*	*95% CI*
**Tobacco risk factors**						
**Age of childhood smoking onset** (years)
Never	**7.47**	**2.28**	**0.67–3.90**	**8.86**	**3.54**	**1.38–5.74**
<15	**-7.90**	**-4.27**	**-6.52 – -1.97**	**-7.71**	**-3.34**	**-6.08 – -0.52**
≥15	Ref	Ref		Ref	Ref	
**Current smoking**						
Never or former	Ref	Ref		Ref	Ref	
Current	**-15.37**	**-4.14**	**-5.99 – -2.26**	**-15.86**	**-7.10**	**-9.33 – -4.82**
**Pack-years smoking**						
For each additional 10 pack-years	**-1.29**	**-0.37**	**-0.54 – -0.20**	**-1.17**	-0.13	-0.33–0.08
**Secondhand smoke exposure**						
For each additional 5 hours per week	**-1.19**	**0.36**	**-0.55 – -0.18**	**-1.55**	**-0.49**	**-0.76 – -0.21**
**Sociodemographic factors**						
**Age** (years)						
40–49	Ref	Ref		Ref	Ref	
50–59	**-3.55**	**-2.07**	**-3.52 – -0.60**	**-3.75**	**-2.69**	**-4.76 – -0.57**
60–69	**-3.90**	**-2.50**	**-4.18 – -0.79**	-0.44	0.54	-1.63–2.75
70–79	**-4.35**	**-3.04**	**-5.15 – -0.88**	**3.34**	**4.20**	**1.46–7.01**
≥80	**-7.50**	**-3.71**	**-7.11 – -0.20**	0.30	3.33	-0.62–8.13
**Sex**						
Female	**-2.16**	**-1.60**	**-2.98 – -0.19**	**-3.00**	**-2.42**	**-4.16 – -0.66**
Male	Ref	Ref		Ref	Ref	
**Race**						
White	Ref	Ref		Ref	Ref	
Black	**-3.78**	-0.42	-2.49–1.69	-0.57	**3.38**	**1.25–5.55**
Other or multiracial	1.27	-1.13	-4.15–1.57	2.48	1.07	-2.82–5.12
**Annual income** (1000 US$)						
≥100	Ref	Ref		Ref	Ref	
50 to <100	**-4.88**	**-2.22**	**-4.14 – -0.28**	**-4.41**	**-2.99**	**-5.15 – -0.77**
25 to <50	**-11.84**	**-7.28**	**-9.46 – -5.04**	**-11.10**	**-8.70**	**-11.37 – -5.96**
<25	**-19.92**	**-13.60**	**-16.16 – -10.95**	**-18.46**	**-14.05**	**-17.06 – -10.93**
Refused/don’t know	**-9.22**	**-4.88**	**-8.22 – -1.41**	**-5.85**	**-4.51**	**-8.32 – -0.55**
**Education level**						
<HS or GED	Ref	Ref		Ref	Ref	
High school diploma	**6.79**	2.62	-0.48–5.82	**9.04**	**4.42**	**0.84–8.13**
Some college or associate’s	**11.26**	**4.19**	**1.74–6.71**	**10.46**	**3.92**	**0.79–7.15**
Bachelor’s or higher	**21.76**	**8.51**	**5.40–11.72**	**19.58**	**6.46**	**2.88–10.17**
**Residence**						
Urban	Ref	Ref		Ref	Ref	
Non-urban	**-2.46**	-0.18	-1.78–1.45	-1.57	0.76	-0.89–2.45

a Patient-Reported Outcomes Measurement Information System Global Physical Health (GPH) and Global Mental Health (GMH) scales.

b We first calculated the inverse of each outcome (1/GPH or 1/GMH) prior to conducting regression models. We then estimated the independent association between childhood smoking and GPH and GMH separately using multivariable weighted gamma-distributed GLMs, adjusting for covariates (all covariates are listed in the table) as described above. For ease of interpretation in terms of the original outcome scales, coefficients from regression models were inverse exponentiated and converted to percentages for reporting. Estimates for all covariates in this weighted analysis are included in the table and represent the percent difference in the outcome associated with the level of the covariate relative to the reference category, with positive values indicating a percent increase and negative values indicating a percent decrease. Bolded values denote statistical significance, p<0.05.

### Multivariable analyses

In the multivariable analysis (Table 2), childhood smoking was associated with -4.27% (95% CI: -6.52 – -1.97) and -3.34% (95% CI: -6.08 – -0.52) decrements on the GPH and GMH outcomes respectively, compared to -4.14% (95% CI: -5.99 – -2.26) and -7.10% (95% CI: -9.33 – -4.82), respectively, for current smoking (compared to never/former smoking). The childhood smoking association was about the same size as the decrement associated with a one-point change in annual income category, for example, comparing U$ 50000 to <100000 with ≥100000 (-2.22%; 95% CI: -4.15 – -0.28) for GPH and -2.99% (95% CI: -5.15 – -0.77) for GMH. Cigarette pack-years were also independently associated with poorer GPH (-0.37%; 95% CI: -0.54 – -0.20), whereas secondhand smoke exposure was independently associated with poorer GPH (-0.36%; 95% CI: -0.55 – -0.18) and GMH (-0.49%; 95% CI: -0.76 – -0.21).

### Sensitivity analysis

As shown in Table 3, childhood smoking retained a significant association with GPH (-2.62%; 95% CI: -4.95 – -0.24), but not GMH (-2.11%; 95% CI: -4.96–0.82) after further adjustment for COPD and other comorbidities, compared to a later onset in cigarette smoking. Having a diagnosis of COPD was also associated with lower scores for both GPH (-8.10%; 95% CI: -10.22 – -5.93) and GMH (-7.67%; 95% CI: -10.34 – -4.91). Approximately one-third of the effect of childhood smoking on GPH (38.6%) and GMH (36.8%) was found to be mediated by COPD diagnosis and other comorbidities.

**Table 3:** Sensitivity of the findings to further adjustment for tobacco-related disease and substitution of smoking duration for cigarette pack-years, a cross-sectional analysis of Wave 5 (2018–2019), Population Assessment of Tobacco and Health Study for adults aged ≥40 years (N=7056), restricted use files analyzed 2024 (estimates reflect percent difference compared to reference)

*Variables*	*Global physical health*			*Global mental health*		
	*Percent difference*			*Percent difference*		
	*Unadjusted*	*Adjusted*	*95% CI*	*Unadjusted*	*Adjusted*	*95% CI*
**Sensitivity analysis adding disease comorbidities (N=7006)**						
**Age of childhood smoking onset** (years)						
Never	**7.47**	0.40	-0.97–1.78	**8.86**	1.91	-0.05–3.91
<15	**-7.90**	**-2.62**	**-4.95 – -0.24**	**-7.71**	-2.11	-4.96–0.82
≥15	Ref	Ref		Ref	Ref	
**Chronic obstructive pulmonary disease** (12 missing values)						
No	Ref	Ref		Ref	Ref	
Yes	**-18.80**	**-8.10**	**-10.22 – -5.93**	**-17.13**	**-7.67**	**-10.34 – -4.91**
**Comorbidity index** (43 missing values)						
For each additional condition	**-5.31**	**-4.34**	**-4.82 – -3.85**	**-3.84**	**-3.32**	**-3.92 – -2.72**
**The proportion of effect mediated for age of smoking onset <15 years** (%)		38.6			36.8	
**Ratio of total to direct effect for age of smoking onset <15 years** (%)		163.0			158.3	
**Sensitivity analysis substituting smoking duration (N=7056)**						
**Age of childhood smoking onset** (years)						
Never	**7.47**	-0.76	-2.92–1.45	**8.86**	1.54	-1.12–4.27
<15	**-7.90**	**-3.79**	**-6.00 – -1.53**	**-7.71**	**-2.91**	**-5.63 – -0.12**
≥15	Ref	Ref		Ref	Ref	
**Current smoking**						
Never or former	Ref	Ref		Ref	Ref	
Current	**-15.37**	-1.66	-4.12–0.85	**-15.86**	**-5.56**	**-8.31 – -2.72**
**Smoking duration**						
For each additional 10 years	**-3.43**	**-1.91**	**-2.87 – -0.93**	**-3.55**	**-1.12**	**-2.07 – -0.16**

We first calculated the inverse of each outcome (1/GPH or 1/GMH) prior to conducting regression models. We then estimated the independent association between childhood smoking and GPH and GMH separately using multivariable weighted gamma-distributed GLMs, adjusting for covariates as described above. For ease of interpretation in terms of the original outcome scales, coefficients from regression models were inverse exponentiated and converted to percentages for reporting. Estimates for all covariates in this weighted analysis are included in the table and represent the percent difference in the outcome associated with the level of the covariate relative to the reference category, with positive values indicating a percent increase and negative values indicating a percent decrease. Bolded values denote statistical significance, p<0.05. Both models also adjust for age, sex, race, income, education level, residence, and secondhand smoke exposure. The first model also includes adjustment for current smoking and pack-years of smoking. The proportion of effect mediated and ratio of total to direct effect presented for the model adjusting for comorbidities are based on Table 2’s adjusted model estimates that did not include comorbidities. The proportion of effect mediated was calculated as [-4.27-(-2.62)]/-4.27 for physical health and [-3.34-(-2.11)]/-3.34 for mental health. The ratio of total to direct effect was calculated as -4.27/-2.62 for physical health and -3.34/-2.11 for mental health.

Substitution of smoking duration for pack-years of smoking had little influence on estimates for childhood smoking; however, the substitution resulted in confounding of the association between current smoking and GPH.

## DISCUSSION

In this sample of older US adults, childhood smoking was associated with poorer global physical and mental health in adulthood, extending previous studies that have found HRQOL associations with current smoking^3,6,7,21-28^, heaviness of current smoking^4^, pack-years of smoking^5^, secondhand smoke exposure^10,11^, and improvements with reducing^29^ or quitting^9,28,30,31^ smoking. The association or childhood smoking was similar in magnitude to current smoking (compared to never/former smoking) and was largely unaffected by how smoking history was measured. The association was as strong as other well-established factors like earnings^32^.

The strength of the association for GMH was attenuated with further adjustment for comorbid diseases, suggesting that smoking-related diseases and their symptoms were important mediators of the association. However, adjusting for comorbid diseases only partially explained decrements in GPH and GMH. Although partial mediation could be explained by inadequate measurement of all the disease processes that cigarette smoking sets in motion, it is also possible that other mechanisms explain it. For example, it is well established that nicotine has unique effects on neurodevelopmental processes during adolescence^33^, processes that not only affect propensity for nicotine addiction, but could also affect other neural networks associated with memory and cognition^34^, networks employed in the very perception of HRQOL.

Current smoking, smoking duration, and secondhand smoke exposure each retained an independent association with global health measures, associations that would be additive if taken together. Thus, the effect of lifetime smoking on HRQOL may have been underestimated by prior studies that addressed any of these established risk factors by themselves. For example, one study that examined current smoking status on the 36-item Short-Form Health Survey questionnaire (SF-36) found that differences ‘were not large’, being of the order of 0.2–0.5 standard deviation^22^. More work is needed to assess the full scope of cigarette smoking through the life course on HRQOL in older adults.

### Strengths and limitations

This study had many strengths, a nationally representative US sample, choice of a well-validated measure of global health that includes a physical and mental component^17,35^, testing of the associations between a novel smoking-descriptor (childhood smoking) and poor HRQOL, adjustment for many other aspects of cigarette smoking that also independently relate to HRQOL, and a sensitivity analysis that appropriately considers smoking-related disease measures as mediators rather than independent risk factors. The findings add further support for the World Health Organization’s Framework Convention on Tobacco Control guidelines aimed at reducing childhood smoking, including smoke-free policies (Article 9), plain packaging (Article 11), public-awareness campaigns aimed at teens (Article 12), a ban on tobacco advertising (Article 13), and a prohibition on sale of tobacco to anyone aged <18 years (Article 16)^36^.

The study also has limitations. This is a complete case analysis and did not use advanced methods to address item non-response. Future studies could apply mediational analysis to formally test the degree to which the association between tobacco risk and HRQOL is mediated through tobacco-related disease. As with any observational study, there could be unmeasured confounders that explain the relationship between childhood smoking and HRQOL. Since it is the first to find an association between childhood smoking and HRQOL in older adults, the finding requires replication and may not generalize across populations. The main finding was based on recall of age of onset of ‘regular’ smoking; it is possible that perceptions of smoking onset were biased in those with lower HRQOL. This is bound to be a persistent problem, as longitudinal studies that determined smoking onset during adolescence are unlikely to follow participants into late adulthood, when HRQOL determinations were made.

## CONCLUSIONS

This study suggests that initiating cigarette smoking early during adolescence is associated with reductions in HRQOL in later adulthood. Although much of this association is explained by the diseases caused by smoking, there may also be long-term consequences beyond disease that relate to how smoking affects development during adolescence. Finally, multiple descriptors of life-course cigarette smoking (current smoking, pack-years of smoking, and secondhand smoke exposure) each had modest additive associations with quality-of-life decrements, suggesting a larger association than has been previously reported.

## Supplementary Material



## Data Availability

The data supporting this research are available from the following source: https://doi.org/10.3886/ICPSR36231.v21
