# Supplementary material for: Childhood cigarette smoking is associated with health-related quality of life in older US adults
Source: Tob Induc Dis. 2025 Jul 23;23:10.18332/tid/204009. doi: 10.18332/tid/204009 (PMC12285645; doi:10.18332/tid/204009)
Supplement: Supplementary file 1 [file TID-23-97-s1.pdf]

Appendix Table. Sample distribution for PROMIS global physical health and global mental health scores, Population Assessment of Tobacco and Health, adults aged 40 and over (N=7,056)

		Physical health	Mental health
Mean		14.8	14.5
SD		3.1	3.8
Percentile			
	1	7.0	5.0
	5	9.0	8.0
	10	10.0	10.0
	25	13.0	12.0
	50	15.0	15.0
	75	17.0	17.0
	90	18.0	19.0
	95	19.0	19.0
	99	20.0	20.0

Appendix Figure 1. Flow diagram for study sample.

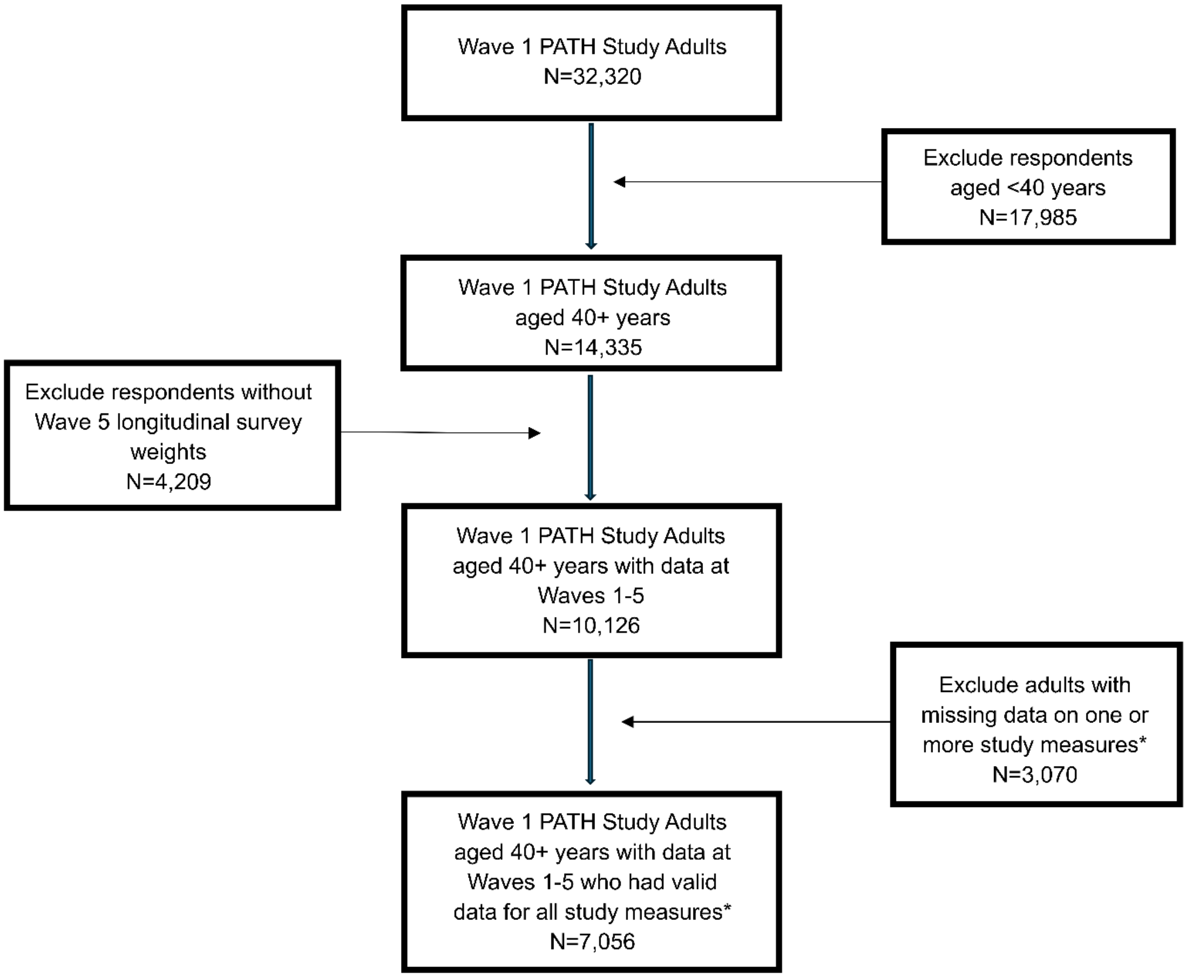

*The item non-response rate was 4.7%.

Appendix Figure 2. Lowess smoothed plot showing the relation between age started smoking regularly and chronic obstructive pulmonary disease (COPD) prevalence from the Population Assessment of Tobacco and Health Study.[1]

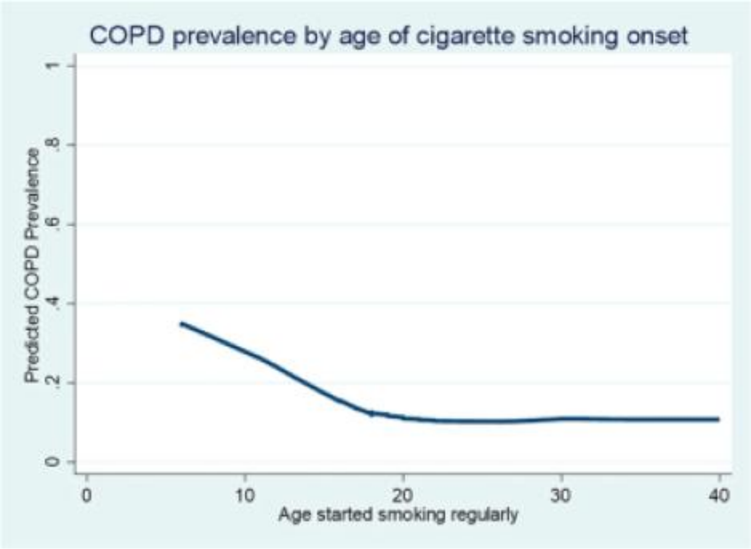

[1] Taken from: Sargent, J. D., Halenar, M., Steinberg, A. W., Ozga, J., Tang, Z., Stanton, C. A., & Paulin, L. M. (2023). Childhood Cigarette Smoking and Risk of Chronic Obstructive Pulmonary Disease in Older U.S. Adults. Am J Respir Crit Care Med, 208(4), 428-434.

© 2025 Ozga J.E. et al.
